# Association Between Self-Reported Pornographic Consumption Habits and Anxiety and Depression: A Systematic Review and Meta-Analysis

**DOI:** 10.3390/jcm15135030

**Published:** 2026-06-27

**Authors:** Jia Yang Tan, Chen Ee Low, Hon Jen Wong, Isaac Yongjie Sim, Charmaine Yong Ching Lee, Hong Ruey Ding, Sean Loke, Cyrus Su Hui Ho

**Affiliations:** 1 Yong Loo Lin School of Medicine, National University of Singapore, Singapore 119077, Singapore; 2 Department of Psychological Medicine, Yong Loo Lin School of Medicine, National University of Singapore, Singapore 119077, Singapore; 3 Department of Psychological Medicine, National University Hospital, Singapore 119074, Singapore

**Keywords:** pornography, pornography addiction, depression, anxiety, mental health

## Abstract

**Objectives:** This study aims to investigate the association between self-reported pornographic consumption habits and anxiety and depression. **Methods:** We systematically searched PubMed, Embase, Cochrane, and PsycINFO for all studies that examined anxiety and depression across consumption habits in pornographic content consumers (PCCs). PRISMA reporting guidelines were followed. Random effects meta-analyses were conducted for the primary analysis using correlation coefficients (CORs) and standardised mean differences (SMDs). A synthesis without a meta-analysis approach was further performed to examine factors affecting pornography consumption. **Results:** A total of 19 studies were included. The overall meta-analysis indicated significant positive correlations between pornographic consumption and both anxiety (COR = 0.16; 95% CI: 0.08; 0.25) and depression (COR = 0.24; 95% CI: 0.15; 0.32). Exploratory study-level subgroup analyses suggested that problematic consumption or addiction, but not general consumption, was associated with anxiety and depression. A stronger anxiety correlation was also observed in samples with a mean age ≤25 years compared with older users (COR = 0.22 vs. COR = 0.09; *p* = 0.0321). Systematically reviewing the studies showed that male gender and younger age significantly increased pornographic content consumption. **Conclusions:** The study found significant positive correlations between pornographic consumption and both anxiety and depression, with an increased effect on younger users. These findings suggest that pornography consumption habits may be important to psychosocial assessment, although substantial heterogeneity, residual confounding, and the observational nature of the evidence preclude causal inference.

## 1. Introduction

The consumption of pornographic content has become increasingly prevalent in today’s digital age, with a growing number of pornographic content consumers (PCCs) accessing such material via smart devices and the Internet. Recent evidence suggests that pornography consumption is widespread across diverse sociocultural settings. For example, a national population-based probability survey [1] conducted on American adults found that 94% of men and 87% of women reported lifetime pornography consumption, beginning on average in their teenage years. Studies from Southeast Asia [2] and Arab countries [3] have demonstrated substantial pornography exposure among young and adult populations, with younger age and male sex associated with greater usage. In the International Sex Survey, which included 82,243 participants from 42 countries across five continents, the estimated risk of problematic pornography consumption ranged from 3.2% to 16.6% [4,5]. This trend has been further amplified by recent factors, including increased Internet usage and the COVID-19 pandemic [6]. Although pornography consumption is often viewed as recreational or harmless, emerging evidence suggests that frequent, compulsive, or problematic use may worsen mental health [7]. Some neurobiological models have proposed that repeated exposure to hyperstimulating sexual content may be associated with changes in the brain’s reward circuitry, leading to depressive features of anhedonia or a blunted affect [8]. Psychologically, the distorted depiction of sex, relationships, and body images in pornography can generate unrealistic expectations and feelings of inadequacy [9].

Simultaneously, mental health conditions such as anxiety and depression have become pressing public health issues in the 21st century [10,11,12,13]. They affect more than 580 million people globally, according to the World Health Organisation in 2015 [14], with young adults and adolescents, the largest group of PCCs [15], being disproportionately impacted. Loneliness [16], excessive digital exposure [17], and evolving social norms around sex and relationships [18] have all contributed to the growing global mental health burden. Loneliness is increasingly recognised as a psychosocial risk factor for poorer mental health, as reduced social connectedness may increase emotional distress and vulnerability to anxiety and depressive symptoms [19]. In this context, pornography may be used as an easily accessible substitute for intimacy or as a maladaptive coping mechanism for unmet emotional and relational needs. Problematic pornography consumption may not only manifest from these stressors but also further reinforce them [20]. While individual studies have found pornography consumption to distort perceptions of oneself [21] and one’s relationships [22], the broader psychological consequences, especially their magnitude and consistency across populations, remain understudied in the scientific literature.

To the best of the authors’ knowledge, this study is the first systematic review and meta-analysis to investigate the effects of pornographic consumption on anxiety and depression. By compiling data from multiple studies and performing subgroup analyses, the study also examined relevant prognostic factors that affect pornography consumption. Such an understanding would be critical for reforming public education and guiding interventional methods for those at risk of the psychological complications of pornography usage.

## 2. Methods

This systematic review was conducted based on the Preferred Reporting Items for Systematic Reviews and Meta-Analyses (PRISMA) guidelines and registered with PROSPERO (CRD420251086306).

A literature search was performed across four major databases: PubMed, Embase, Cochrane, and PsycINFO. These databases were selected for their broad coverage of the biomedical, clinical, psychological, psychiatric, and trial-related literature relevant to problematic pornography consumption and mental health outcomes. PubMed and Embase were searched for biomedical and clinical studies, the Cochrane Library for trial and review evidence, and PsycINFO for the psychology- and psychiatry-focused literature. The search strategy integrated terms related to “Pornography”, “Depression” and “Anxiety”. Relevant subject headings with appropriate truncations were utilised using specific database-controlled vocabulary, and synonyms with the appropriate truncations were used. Examples of search strategies for PubMed and Embase are provided in Supplementary Table S1.

**Figure 1:**
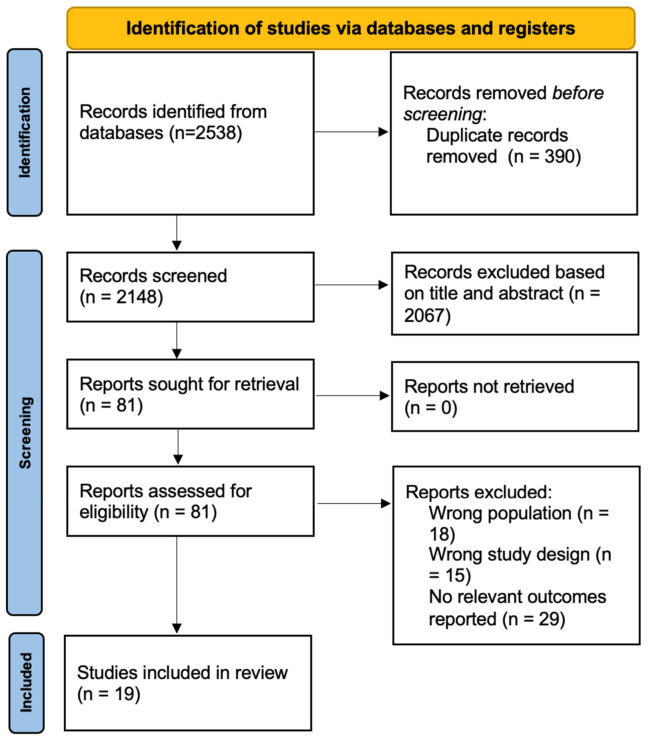
PRISMA flowchart.

Three reviewers independently screened the titles and abstracts of the studies to determine eligibility. Discrepancies between reviewers were resolved by a fourth independent reviewer. All studies examining anxiety and depression using validated psychometric instruments in PCCs were included if published in peer-reviewed English-language journals from inception to 2 July 2025. Studies were excluded if they were non-empirical, conference abstracts or grey literature. The study selection process is shown in Figure 1.

Extracted data included participant demographics, correlation, anxiety and depression scores, religiosity and self-esteem. The primary exposure was broadly defined as pornography-related consumption habits. Where possible, studies were classified by habits: general consumption, problematic or compulsive use, and pornography addiction or self-perceived addiction. Statistical analyses were performed using R (version 4.1.0) with the meta and metafor packages. A two-sided *p*-value of less than 0.05 was considered statistically significant. The primary outcomes were correlation, anxiety and depression scores. Correlation scores were pooled using the metacor function and interpreted using Cohen’s guidelines [23]. Anxiety and depression scores were pooled as standardised mean differences (SMDs) using the metacont function to compare the severity of these scores between male gender as the exposed group and female gender as the control group.

Subgroup analyses were conducted if there were sufficient studies. The subgroup analyses conducted were stratified by type of consumption (problematic consumption or addiction vs. general consumption), mean patient age, (≤25 vs. >25 years), proportion of male participants (<50% vs. ≥50%), world bank income classification (low, lower-middle, upper-middle, or high income) [24], and World Health Organisation (WHO) region [25]. If there were insufficient studies for subgroup analyses, a synthesis without a meta-analysis approach was performed [26,27,28,29].

Between-study heterogeneity was quantified using I^2^ and τ^2^ statistics [30]. An I^2^ value below 30% was treated as low heterogeneity, 30% to 60% as moderate, and above 60% as substantial.

Sensitivity analyses were conducted by leave-one-out analyses to assess the influence of individual studies on the overall result. The risk of bias for each included study was assessed using the Joanna Briggs Institute (JBI) Critical Appraisal Tool [31]. Cross-sectional studies were assessed using the analytical cross-sectional checklist, while longitudinal cohort studies were assessed using the cohort checklist.

## 3. Results

Out of the 2538 reports identified from the databases (Figure 1), we included a total of 19 studies [7,32,33,34,35,36,37,38,39,40,41,42,43,44,45,46,47,48,49] evaluating depression and anxiety in PCCs. The remaining 2519 studies were excluded after removing duplicates and irrelevant studies with no relevant outcomes, wrong study design and wrong population.

The main characteristics of the studies are reported in Table 1. The studies’ sizes ranged from 280 to 1639 participants, with a total of 17,529 PCCs in the final analysis. Seven of the studies were from the United States of America [33,34,36,42,45,46,47]; two studies were from Croatia [38,48]; two studies recruited online participants [37,40]; and one study each was from Italy [7], the Netherlands [35], Pakistan [32], China [39], Sweden [41], the United Kingdom [43], Israel [44] and Egypt [49]. The mean age of PCCs at the time of the study ranged from 15.2 to 41.1 years old. Six studies [7,32,33,36,37,49] investigated the correlation between pornography consumption and anxiety. Ten studies [7,32,33,35,36,37,39,40,41,49] investigated the correlation between pornography consumption and depression. Three studies [32,33,34] evaluated the anxiety scores between males and females. Five studies [32,33,39,40,41] evaluated the depression scores between males and females. The included studies assessed different pornography-related concepts, including general pornography consumption, compulsive use and pornography addiction, which are related but not equivalent clinically. For consistency, this review used the umbrella term “pornography consumption” while recognising the conceptual heterogeneity between the studies. Classification of studies according to the pornography-related construct assessed can be found in Supplementary Table S2.

### 3.1. Meta-Analysis of the Correlation of Pornographic Consumption on Anxiety and Depression

**Figure 2:**
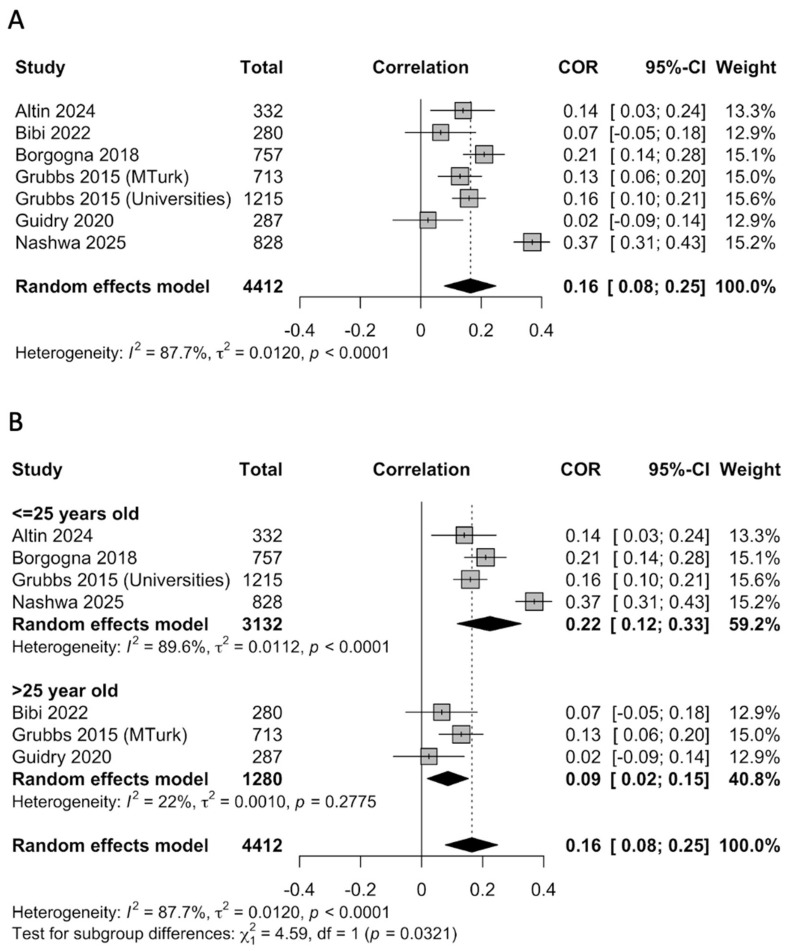
Pooled correlation scores between pornography consumption and anxiety (**A**) stratified by age (**B**) [7,32,33,36,37,49].

The meta-analysis of six studies (Figure 2A) [7,32,33,36,37,49] indicated a positive correlation between pornography consumption and anxiety, with a correlation of 0.16 (95% CI: 0.08; 0.25). When subgrouped by type of consumption habits (Supplementary Figure S1), there was a significant correlation of 0.19 (6 cohorts, 95% CI: 0.07; 0.30) amongst those with pathological consumption, while there was no significant correlation amongst those with general consumption (1 cohort, 95% CI: −0.09; 0.14). Subgroup meta-analyses showed that pornographic content consumers whose age ≤ 25 years old had a statistically significant stronger correlation between pornographic consumption and anxiety with a correlation of 0.22 (95% CI: 0.12; 0.33) compared to those age > 25 years old with a correlation of 0.09 (95% CI: 0.02; 0.15) (test for subgroup differences, *p* = 0.0321) (Figure 2B). Subgroup meta-analyses (Supplementary Table S3) showed no significant differences (test for subgroup differences, *p* > 0.05) in WHO region, income levels, percentage of males or scale used to assess for anxiety.

**Figure 3:**
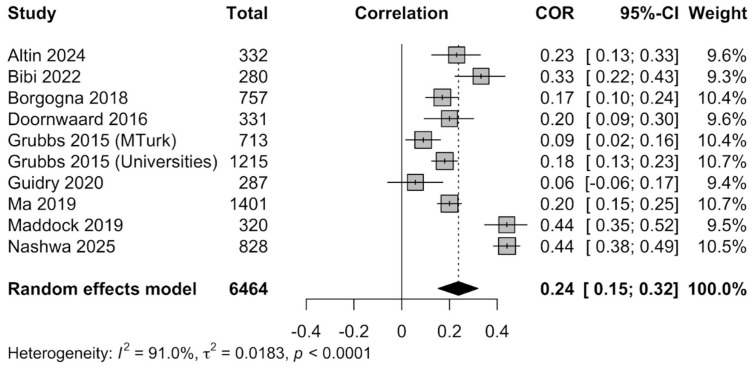
Pooled correlation scores between pornography consumption and depression [7,32,33,35,36,37,39,40,49].

The meta-analysis of nine studies (Figure 3) [7,32,33,35,36,37,39,40,49] indicated a positive correlation between pornography consumption and depression, with a correlation of 0.24 (95% CI: 0.15; 0.32). When subgrouped by type of consumption habits (Supplementary Figure S2), there was a significant correlation of 0.26 (eight cohorts, 95% CI: 0.15; 0.37) amongst those with pathological consumption, while there was no significant correlation amongst those with general consumption (two cohorts, 95% CI: −0.65; 0.78). Subgroup meta-analyses showed no significant differences in age, percentage of males or WHO region. However, subgroup analysis revealed significant differences (test for subgroup differences, *p* < 0.0001) in the types of depression scale used and income levels (Supplementary Table S4).

**Figure 4:**
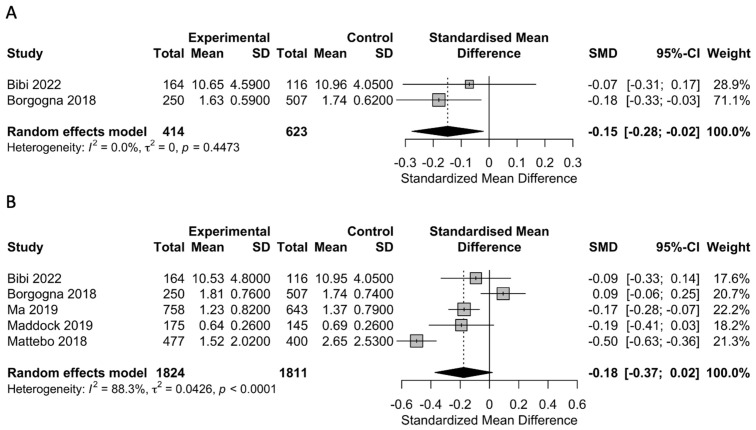
Pooled standardised mean differences in anxiety (**A**) [32,33] and depression (**B**) [32,33,39,40,41] scores between male and female pornography content consumers.

Meta-analyses of two studies (Figure 4A) [32,33] found that male PCCs have lower anxiety scores compared to females, with a statistically significant pooled SMD of −0.15 (95% CI: −0.28; −0.02). Meta-analyses of five studies (Figure 4B) [32,33,39,40,41] found that male PCCs had lower depression scores than female PCCs, although this difference was not statistically significant, with a pooled SMD of −0.18 (95% CI: −0.37; 0.02) (Supplemental Table S5). The SMD for Mattebo et al. [41] was notably much lower than the rest, likely because they hypothesised that pornography consumption was more socially acceptable for male users, posing minimal threat to their mental health.

### 3.2. Systematic Review of the Prognostic Factors Affecting Pornographic Consumption

The study examined the prognostic factors affecting pornographic consumption. These factors include demographic variables such as gender, age, religiosity and self-esteem.

#### 3.2.1. Age

Five studies [34,42,43,44,45] evaluated the association between age and pornographic consumption (Supplementary Table S6). Three studies [43,44,45] found that a younger age is significantly associated with higher pornographic consumption. Noel and Camilleri et al. [34,42] found a significant increase in pornography usage in consumers of older ages.

#### 3.2.2. Gender

Eight studies [39,40,41,42,43,44,45,48] explored the link between gender and pornographic consumption (Supplementary Table S7). All eight studies [39,40,41,42,43,44,45,48] found that males are significantly associated with higher pornographic consumption.

#### 3.2.3. Religiosity

Two studies [39,40] investigated the association between religiosity and pornographic consumption (Supplementary Table S8). Both studies [39,40] found no significant difference in pornographic consumption between consumers who are either religious or non-religious.

#### 3.2.4. Self-Esteem

Two studies [35,38] explored the association between self-esteem and pornographic consumption (Supplementary Table S9). Doornward et al. [35] found significantly increased pornographic use in those with low global self-esteem, while Kohut et al. [38] found no significant association between self-esteem and pornographic consumption.

### 3.3. Risk-of-Bias and Publication Bias

The quality of the 19 studies [7,32,33,34,35,36,37,38,39,40,41,42,43,44,45,46,47,48,49] was assessed using Joanna Briggs’ Institute Critical Appraisal tool and is presented in Supplementary Tables S10 and S11. Overall, there was no significant risk of bias identified. Sensitivity analysis using outlier and leave-one-out analyses identified Maddock et al. (Supplementary Figures S3–S8) [40] as an outlier for depression, possibly due to a larger proportion of religious participants in which pornography consumption may cause guilt and moral conflict [20], and Nashwa et al. [49] for anxiety, possibly because Egypt has strong religious taboos surrounding pornography, amplifying psychological distress after its consumption [50].

## 4. Discussion

This study is the first systematic review and meta-analysis that sought to investigate the association between pornographic consumption habits and anxiety and depression. The results demonstrated significant positive correlations between pornographic consumption and both anxiety and depression. However, these findings should be interpreted in the context of substantial heterogeneity and the observational nature of the included studies. Subgroup analyses found that PCCs aged ≤ 25 years showed a stronger correlation with anxiety compared to older users. Male PCCs have significantly lower anxiety scores compared to females, but there was no significant gender difference observed for depression scores. The systematic review highlighted that male gender and younger age significantly increased pornographic content consumption. Most importantly, only those with pathological consumption, defined as problematic use or addiction, had a significant correlation with anxiety and depression.

The authors found a positive correlation between pornographic consumption and anxiety, which is consistent with the findings of existing studies [20,51]. Pornography use may be associated with shame or guilt in some individuals, particularly where use conflicts with personal, cultural, or religious values [52]. However, these associations may also reflect moral incongruence, stigma, secrecy, or pre-existing psychological distress rather than a direct effect of pornography consumption itself. Its use can perpetuate cycles of shame and guilt that can be difficult to break due to fears of being discovered and judged by others, manifesting as anxiety symptoms. This is particularly so for individuals when their sexual behaviours clash with their personal values and beliefs [20] that pornography is morally objectionable and harmful [53]. For example, religiously affiliated individuals are more likely to have stronger negative attitudes towards pornography consumption and express a moral disapproval of it, a view rooted in conservative religious beliefs [54]. Hence, cultural and religious differences may influence the observed association. Additionally, the unrealistic portrayals in pornographic scenes can create pressure for consumers to perform in specific ways, creating performance anxiety in real-world sexual encounters as they compare themselves to what they observe in pornography [55].

Subgroup analysis revealed that younger PCCs aged less than 25 years showed a stronger correlation with anxiety compared to older users. This finding is consistent with the current understanding of developmental psychology, as adolescence and young adulthood are key periods in personal identity development [56,57,58] and exploration in areas such as love [59], including establishing intimate connections with others [60]. Hence, pornographic consumption during this period, when they are more impressionable, may contribute to larger effects on their beliefs and behaviours. Neurodevelopmentally, as their pre-frontal cortex, which is critical for decision making and emotional control [61], has yet to fully mature [62], they might be more vulnerable to the ill effects of compulsive pornographic consumption and its associated anxiety. Furthermore, they lack the maturity to critically evaluate these unrealistic depictions of sex and relationships shown in pornographic content [63]. The systematic review also identified younger age as being significantly associated with more pornographic content consumption [64], likely due to greater digital fluency [65] and sexual curiosity [66], which may contribute to the higher anxiety correlation observed, as they are more susceptible to the ill effects of pornography consumption with greater exposure. Collectively, these factors may explain why younger consumers are more affected and highlight the need for targeted age-specific interventions and education aimed at promoting healthy relationships and sexual intimacy, as well as the potential psychological risks of pornography consumption during their important formative years of personal development and exploration.

The authors also found a positive correlation between pornographic consumption and depression, which aligns with much evidence from the existing literature. For example, Koob et al. [67] found that the overstimulation of the body’s reward system from watching pornographic content can cause withdrawal symptoms when such behaviour is stopped for a period, which include depression and cognitive impairment. A study conducted in California that involved 1864 young adults also found an association of depressive symptoms with frequent pornographic viewing [68]. This can be explained through several mechanisms. From a neurobiological point of view, chronic pornographic use may cause the disruption of the dopaminergic pathways in the brain’s reward system [69]. Over time, this prolonged exposure may contribute to tolerance, where PCCs will require increasingly novel or extreme stimuli to experience the same level of pleasure. When increasingly more pleasurable activities start to become less satisfying, this may potentially be associated with anhedonia [70] and emotional numbness [71], which are all hallmark features of depression [72]. Psychologically, pornographic consumption can create unrealistic expectations about sexual behaviour and body image. Unrealistic and idealised sexual scenarios portrayed in pornographic content can distort one’s view of real-life sexual relationships and intimacy [73]. Pornographic actors also typically have the “perfect” body types, which can potentially evoke feelings of inadequacy and low self-esteem amongst PCCs when they start comparing their own bodies to theirs [74]. A study conducted in Israel involving 726 heterosexual and sexual minority men found that problematic pornography consumption was associated with greater social body comparison and, as a result, more negative perceptions of their own body image [9].

The analyses revealed that male PCCs show significantly lower anxiety scores compared to females while showing no significant gender difference in depression scores. This can be due to current societal norms and cultural attitudes towards pornography, where consuming pornography is more socially acceptable for men than for women [75]. Conversely, in many cultures, female sexual desire and pornography usage are often viewed as socially deviant, disgraceful and indecorous [76]. Hence, males can consume pornography with fewer of the internal conflicts previously discussed and are protected from experiencing the same levels of anxiety compared to females. In addition, pornography may be more readily adopted by men as a coping tool because male sexuality is often socially constructed as more visually driven and permissive of pornography consumption [77], making pornography a more accessible and culturally sanctioned means of emotion regulation. In contrast, women may be less likely to use pornography for coping due to greater stigma, internalised shame, and lower perceived acceptability of consumption, which may instead increase anxiety when problematic use occurs. Secondly, multiple studies also reported that men often engage in pornography as a form of coping mechanism to manage their negative emotions and alleviate stress [78]. The functional use of pornographic content to alleviate anxiety symptoms can explain the lower anxiety scores compared to females. A study by Rosann et al. that explored gender differences in coping strategies found that women tend to use more emotion-focused coping strategies compared to men when dealing with external stressors in their lives and were also reported to have a higher risk of anxiety-related symptoms [79]. However, reverse causation is also plausible as individuals with anxiety may differ in how they use or report pornography. On the other hand, no significant gender differences in depression scores can be explained by the shared impact of long-term pornography usage on relationship quality, personal body image and self-esteem, as previously discussed. These outcomes are not inherently gender-specific and can impact both males and females to the same extent.

The systematic review identified males as being significantly associated with more pornographic content consumption [80]. Collectively, the findings of higher consumption patterns, together with statistically significantly lower anxiety scores compared to females, need to be carefully interpreted, particularly in the context of public health. The results should not be misconstrued as evidence that pornography consumption is psychologically beneficial to male users, but rather, they highlight the complex interplay between gender roles, individuals’ coping strategies, societal norms and expectations, and psychological outcomes, which requires a nuanced, individualised approach. It is possible that male PCCs experience anxiety relief through pornographic use as an emotional regulation tool [81].

The findings of this study should also be interpreted within the broader framework of problematic digital and behavioural addictions. Similar to other behavioural addictions, PCCs may be linked to emotional dysregulation [82], maladaptive coping behaviours [20], poorer mental well-being and difficulties with interpersonal or prosocial behaviours [41]. Previous studies have additionally associated PCCs with lower relationship [83] and sexual satisfaction [84], suggesting that its psychosocial impact may extend beyond anxiety and depression alone. However, the relationship between PCCs and psychological outcomes should also not be viewed solely through a pathological lens, and protective psychosocial factors may also play an important role. Possible protective factors, such as having a strong support system [85] and higher digital and sexual literacy [77] can reduce the vulnerability to such behaviours and their associated psychological outcomes, as they make individuals less likely to rely on pornography consumption as a mechanism for stress relief and better evaluate unrealistic portrayals of intimacy depicted in pornographic content, respectively.

Given the plausible association between pornographic consumption and symptoms of anxiety and depression, greater clinical awareness may be warranted across both psychiatric and primary care settings. Healthcare providers may consider assessing pornographic consumption as part of a broader psychosocial assessment, particularly amongst patients presenting with high levels of stress, identity-related concerns or maladaptive coping behaviours. Of note, anxiety and depression may themselves contribute to excess stress and thus increased pornographic consumption as a coping mechanism, highlighting the possibility of a bidirectional relationship [20]. Management might therefore require an approach that addresses access to pornography through social support measures, digital and sexual literacy [86], addiction-related interventions such as cognitive behavioural therapy [87], and targeted treatment of the underlying psychiatric symptoms [88,89]. Further primary studies are needed to clarify the association between pornography consumption and anxiety or depression, and to elucidate the contributing and protective factors that could help inform holistic care for these patients.

### Limitations

Several limitations may reduce the applicability of the findings. Firstly, most included studies were observational, and many were cross-sectional, precluding conclusions about temporality or causality. Reverse causation is a major concern, as individuals with pre-existing anxiety, depression, loneliness, low self-esteem, relationship dissatisfaction, or emotional dysregulation may be more likely to use pornography as a coping mechanism. Secondly, the lack of previous psychological assessments prior to the consumption of pornographic content is also a significant limitation. As the findings only reflect their psychological state after pornography usage, we are unable to exclude the possibility that participants may already have anxiety and depression before watching pornography. There is also a possibility that male consumers already have lower anxiety scores independent of pornography usage. Thirdly, the limited number of studies available for subgroup analysis limited the robustness of the results. For example, we initially planned to investigate how consumption of different types of pornographic content can have differential psychological effects but could not proceed due to limited studies. Therefore, longitudinal and prospective studies should assess temporality, baseline mental health, confounding factors, and changes in pornography-related behaviours over time. Fourthly, there was high heterogeneity among the results, with variability in terms of assessment tools used to assess psychological outcomes, countries, income levels and other sociocultural and economic factors. Therefore, the subgroup analyses were constrained by the limited number of studies in each category. Hence, the findings of our subgroup analyses may be considered as primarily exploratory in nature. Importantly, differences in assessment scales across studies had limited direct comparability, as evidenced by significant subgroup differences observed for the correlation between pornography consumption, depression, and depression scores in our analysis. Although this heterogeneity may have influenced the magnitude of the observed associations, the plausible association between pornography consumption and depression still remains an important finding. This limitation also underscores the need for future controlled studies utilizing standardised assessment scales to provide more robust insight. Additionally, most studies assessed pornography consumption on a graded scale and correlated this to their anxiety and depression scores, rather than a binary classification of pornographic users and non-pornographic users. Hence, individuals with little to no pornographic consumption are still included in the analysis, and we are unable to determine psychological outcomes solely based on individuals with high pornographic consumption patterns. Another limitation is the conceptual heterogeneity between studies. While some studies assessed general pornography consumption, others evaluated problematic or compulsive pornography consumption, which may differ clinically and influence the pooled effect sizes and contribute to heterogeneity.

## 5. Conclusions

The study found significant positive correlations between pornographic consumption and both anxiety and depression. PCCs who were less than 25 years old showed a stronger correlation with anxiety compared to older users. Male PCCs have significantly lower anxiety scores compared to females, but there was no significant gender difference observed for depression scores. Anxiety and depression were only associated with pathological use but not general consumption. A systematic review highlighted that male gender and younger age significantly increased pornographic content consumption. Overall, a comprehensive pornographic use assessment may be relevant as part of mental health evaluations in selected individuals facing psychological problems, including subjective distress and functional impairment. In addition, a focus on public education about these associations can be beneficial to the general population. Public health professionals can aim to promote more open and stigma-free conversations about healthy sexual behaviour and its association with the emotional well-being and digital use of individuals.

## Figures and Tables

**Table 1:** Main characteristics of the included studies.

Author	Publication Year	Region of Study	Population Studied	Gender Male (Proportion 0–1)	Age at Study (Mean ± SD)	Total Number of Participants	How Was Pornography Consumption/Addiction Assessed	Anxiety Scale	Correlation Scores Between Anxiety and Pornographic Use	Anxiety Scores for Males (Mean ± SD)	Anxiety Scores for Females (Mean ± SD)	Depression Scale	Correlation Scores Between Depression and Pornographic Use	Depression Scores for Males (Mean ± SD)	Depression Scores for Females (Mean ± SD)
Altin	2024 [7]	Italy	Adults > 18	0.36	23.41 ± 2.84	332	CYPAT	DASS	0.14	NR	NR	DASS	0.23	NR	NR
Bibi	2022 [32]	Pakistan	Adults > 18	0.587	25.4 ± 5.271	280	PCQ and BPS	DASS	0.066	10.65 ± 4.59	10.96 ± 4.05	DASS	0.332	10.53 ± 4.8	10.95 ± 4.05
Borgogna	2018 [33]	USA	University students	0.33	24.13	757	PPUS	DASS	0.21	1.63 ± 0.59	1.74 ± 0.62	DASS	0.17	1.81 ± 0.76	1.74 ± 0.74
Camilleri	2020 [34]	USA	Adults > 18	0.34	20.76	1031	mCIUS	DASS	NR	8	10	DASS	NR	NR	14
Doornwaard	2016 [35]	Netherlands	Adolescent boys	1	15.16 ± 1.31	331	Compulsive Internet Use Scale	NR	NR	NR	NR	Depressive Mood Scale	0.2	NR	NR
Grubbs (MTurk)	2015 [36]	USA	Adults > 18	0.519	30.2 ± 9.9	713	Study-specific 0–12 scale to quantify consumption, CPUI-9 for pornography addiction	GAD-7	0.13	NR	NR	CES-D-10	0.09	NR	NR
Grubbs (Universities)	2015 [36]	USA	Adults > 18	0.672	19.3	1215	Study-specific 0–12 scale to quantify consumption, CPUI-9 for addiction	GAD-7	0.16	NR	NR	CES-D-10	0.18	NR	NR
Guidry	2020 [37]	Online	Adults aged 18–73	0.491	36.75	287	Study-specific 5-point Likert scale to quantify consumption	DASS	0.024	NR	NR	DASS	0.057	NR	NR
Kohut (Zagreb)	2018 [38]	Croatia	Adolescents aged 15–19	0.311	16.1 ± 0.46	643	Study-specific 1–7 scale to quantify consumption	PHQ-4	NR	NR	NR	PHQ-4	NR	NR	NR
Kohut (Rijeka)	2018 [38]	Croatia	Adolescents aged 15–19	0.397	15.9 ± 0.52	1179	Study-specific 1–7 scale to quantify consumption	PHQ-4	NR	NR	NR	PHQ-4	NR	NR	NR
Ma	2019 [39]	China	Grade 7 students	0.541	12.43 ± 0.54	1401	Study-specific 5-point Likert scale to quantify consumption	NR	NR	NR	NR	PHQ-9	0.2	1.23 ± 0.82	1.37 ± 0.79
Maddock	2019 [40]	Online	Adults aged 19–75	0.548	36.26 ± 10.18	320	Excessive Use Subscale of the Problematic Pornography Use Scale and study-specific questions	NR	NR	NR	NR	CESD-R-10	0.44	0.64 ± 0.26	0.69 ± 0.26
Mattebo	2018 [41]	Sweden	Adolescents aged 7–15	0.544	16.25 ± 0.537	877	Study-specific 0–6 scale to quantify consumption	NR	NR	NR	NR	DSRS	M—0.019; F—0.153	1.52 ± 2.02	2.65 ± 2.53
Noel	2023 [42]	USA	Adults aged 18–25	0.55	21.3 ± 2.1	1022	PPCS-6	GAD-7	NR	NR	NR	CES-D-10	NR	NR	NR
Sallie	2021 [43]	United Kingdom	Adults > 18	0.747	28.93 ± 12.46	1344	CYPAT	HADS	NR	NR	NR	HADS	NR	NR	NR
Varod	2024 [44]	Israel	Adults > 18 who can read Hebrew	0.17	25.41 ± 6.54	463	PPCS—Short Version	BSI-A	NR	NR	NR	BSI-18	NR	NR	NR
Weaver	2011 [45]	USA	Internet-using adults	0.481	NA	559	SEMB	NR	NR	NR	NR	CES-D-10	NR	NR	NR
Whitfield	2018 [46]	USA	Adults > 18, cisgender male, self-identified as gay or bisexual, and reported having sex with a man in the past year	1	41.14 ± 12.82	1021	Study-specific questionnaire to quantify consumption	BSI-A	NR	NR	NR	CES-D-10	NR	NR	NR
Willoughby	2019 [47]	USA	Online	0.565	33.56 ± 10.62	1639	Study-specific questionnaire to quantify consumption	NR	NR	NR	NR	CES-D-10	NR	NR	NR
Stulhofer	2019 [48]	Croatia	High school students	0.398	15.9 ± 0.52	1287	Study-specific questionnaire to quantify consumption	PHQ-4	NR	NR	NR	PHQ-4	NR	NR	NR
Nashwa	2025 [49]	Egypt	Nursing students	0.37	20.74 ± 1.7	828	PAST	DASS	0.369	NR	NR	DASS	0.441	NR	NR

Abbreviations: NR, not reported; SD, standard deviation; United States of America, USA; DASS, Depression Anxiety Stress Scale; GAD-7, General Anxiety Disorder-7; PHQ-4, Patient Health Questionnaire-4; HADS, Hospital Anxiety and Depression Scale; BSI-A, Brief Symptom Inventory-Anxiety; CES-D-10, Center for Epidemiological Studies Depression Scale-10 item; PHQ-9, Patient Health Questionnaire-9; CESD-R-10, Center for Epidemiologic Studies Depression Scale Revised-10 item; DSRS, Depression Self-Rating Scale; BSI-18, Brief Symptom Inventory-18; CYPAT, Cyber Pornography Addiction Test; PCQ, Pornographic Craving Questionnaire; BPS, Brief Pornography Screening; PPUS, Problematic Pornography Use Scale; mCIUS, Modified Compulsive Internet Use Scale; CPUI-9, Cyber Pornography Use Inventory-9; PPCS-6, Problematic Pornography Consumption Scale-6; PPCS- Short Version, Problematic Pornography Consumption Scale-Short Version; SEMB, Sexually Explicit Media Use Behavior; PAST, Pornography Addiction Screen Tool.

## Data Availability

All data generated or analysed during this study are included in this article. Further enquiries can be directed to the corresponding author.

## Supplementary Materials

The following supporting information can be downloaded at https://www.mdpi.com/article/10.3390/jcm15135030/s1. PRISMA_2020_abstract_checklist_020526_JYT; PRISMA_2020_checklist_020526_JYT; Table S1: Search Strategy; Table S2: Classification of included studies according to the pornography-related construct assessed and measurement instrument used; Table S3: Subgroup meta-analyses of anxiety correlation observed in pornographic content consumers using the random effect model; Table S4: Subgroup meta-analyses of depression correlation observed in pornographic content consumers using the random effect model; Table S5: Subgroup meta-analyses of depression scores observed in pornographic content consumers between males and females using the random effect model; Table S6: Evaluation of the mediating or confounding effect of age on pornographic consumption and mental health outcomes; Table S7: Evaluation of the mediating or confounding effect of gender on pornographic consumption and mental health outcomes; Table S8: Evaluation of the mediating or confounding effect of religiosity on pornographic consumption and mental health outcomes; Table S9: Evaluation of the mediating or confounding effect of self-esteem on pornographic consumption and mental health outcomes; Table S10: Quality assessment of included cohort studies using the Joanna Brigg’s Institute Critical Appraisal tool; Table S11: Quality assessment of included cross-sectional studies using the Joanna Briggs Institute Critical Appraisal tool; Figure S1: Pooled correlation scores between pornography consumption and anxiety, subgrouped by consumption habits; Figure S2: Pooled correlation scores between pornography consumption and depression, subgrouped by consumption habits; Figure S3: Outlier assessment of studies assessing depression correlation among pornographic content consumers using the random effects model; Figure S4: Leave-one-out assessment of studies assessing depression correlation among pornographic content consumers using the random effects model; Figure S5: Outlier assessment of studies assessing anxiety correlation among pornographic content consumers using the random effects model; Figure S6: Leave-one-out assessment of studies assessing anxiety correlation among pornographic content consumers using the random effects model; Figure S7: Outlier assessment of studies assessing depression scores among pornographic content consumers between males and females using the random effects model; Figure S8: Leave-one-out assessment of studies assessing depression scores among pornographic content consumers between males and females using the random effects model.

## Abbreviation

PCC	Pornographic Content Consumer
