# Supplementary material for: Association Between Self-Reported Pornographic Consumption Habits and Anxiety and Depression: A Systematic Review and Meta-Analysis
Source: J Clin Med. 2026 Jun 27;15(13):5030. doi: 10.3390/jcm15135030 (PMC13362642; doi:10.3390/jcm15135030)

Association between self-reported pornographic consumption habits and anxiety and depression: A systematic review and meta-analysis

Supplementary Table 1: Search Strategy
Supplementary Table 2: Classification of included studies according to the pornography-related construct assessed and measurement instrument used
Supplementary Table 3: Subgroup meta-analyses of anxiety correlation observed in pornographic content consumers using the random effect model
Supplementary Table 4: Subgroup meta-analyses of depression correlation observed in pornographic content consumers using the random effect model
Supplementary Table 5: Subgroup meta-analyses of depression scores observed in pornographic content consumers between males and females using the random effect model
Supplementary Table 6: Evaluation of the mediating or confounding effect of age on pornographic consumption and mental health outcomes
Supplementary Table 7: Evaluation of the mediating or confounding effect of gender on pornographic consumption and mental health outcomes
Supplementary Table 8: Evaluation of the mediating or confounding effect of religiosity on pornographic consumption and mental health outcomes
Supplementary Table 9: Evaluation of the mediating or confounding effect of self-esteem on pornographic consumption and mental health outcomes
Supplementary Table 10: Quality assessment of included cohort studies using the Joanna Brigg's Institute Critical Appraisal tool
Supplementary Table 11: Quality assessment of included cross-sectional studies using the Joanna Briggs Institute Critical Appraisal tool

Supplementary Figure 1: Pooled correlation scores between pornography consumption and anxiety, subgrouped by consumption habits.
Supplementary Figure 2: Pooled correlation scores between pornography consumption and depression, subgrouped by consumption habits.
Supplementary Figure 3: Outlier assessment of studies assessing depression correlation among pornographic content consumers using the random effects model
Supplementary Figure 4: Leave-one-out assessment of studies assessing depression correlation among pornographic content consumers using the random effects model
Supplementary Figure 5: Outlier assessment of studies assessing anxiety correlation among pornographic content consumers using the random effects model
Supplementary Figure 6: Leave-one-out assessment of studies assessing anxiety correlation among pornographic content consumers using the random effects model
Supplementary Figure 7: Outlier assessment of studies assessing depression scores among pornographic content consumers between males and females using the random effects model
Supplementary Figure 8: Leave-one-out assessment of studies assessing depression scores among pornographic content consumers between males and females using the random effects model

Supplementary Table 1: Search Strategy

PubMed - 682

#1	(pornography[MeSH]) OR (problematic pornography[Title/Abstract]) OR (pornography addiction[Title/Abstract]) OR (compulsive pornography use[Title/Abstract]) OR (porn*[Title/Abstract]) OR (sexually explicit material[Title/Abstract]) OR (illicit material[Title/Abstract]) OR (erotic[Title/Abstract]) OR (cyberpornography[Title/Abstract]) OR (cybersex[Title/Abstract]) OR (hypersexuality[Title/Abstract]) OR (compulsive sex[Title/Abstract]) OR (visual sexual stimuli[Title/Abstract]) OR (impulsive sex[Title/Abstract])
#2	(Suicide[Mesh]) OR (Suicide, Attempted[Mesh]) OR (Suicide, Completed[Mesh]) OR (Suicidal Ideation[Mesh]) OR (Suicid*[Title/Abstract]) OR (Self-harm[Title/Abstract]) OR (Self-injur*[Title/Abstract]) OR (deliberate self-harm[Title/Abstract]) OR (parasuicide[Title/Abstract]) OR (self-wounding[Title/Abstract]) OR (self-mutilation[Title/Abstract]) OR (auto-aggression[Title/Abstract]) OR (depression[Mesh]) OR (depressive disorder[Mesh]) OR (anxiety[Mesh]) OR (anxiety disorder*[Mesh]) OR (Schizophrenia[Mesh]) OR (Stress Disorders, Post-Traumatic[Mesh]) OR (Depressive*[Title/Abstract]) OR (Anxi*[Title/Abstract]) OR (Suicid*"[Title/Abstract]) OR (Post-Traumatic*[Title/Abstract]) OR (PTSD[Title/Abstract]) OR (posttraumatic*[Title/Abstract]) OR (Post Traumatic*[Title/Abstract]) OR (Disorder*, Schizophrenic[Title/Abstract]) OR (Schizo*[Title/Abstract]) OR (Psychotic[Title/Abstract]) OR (Psychosis[Title/Abstract])

#1 and #2

Embase - 1437

#1	'pornography'/exp OR 'problematic pornography':ti,ab OR 'pornography addiction':ti,ab OR 'compulsive pornography use':ti,ab OR 'porn*':ti,ab OR 'sexually explicit material':ti,ab OR 'illicit material':ti,ab OR 'erotic':ti,ab OR 'cyberpornography':ti,ab OR 'cybersex':ti,ab OR 'hypersexuality':ti,ab OR 'compulsive sex':ti,ab OR 'visual sexual stimuli':ti,ab OR 'impulsive sex':ti,ab
#2	('depression' OR 'anxiety' OR 'anxiety disorder' OR 'posttraumatic stress disorder' OR ‘suicide’ OR ‘suicide ideation’ OR ‘schizophrenia’ OR ‘psychosis’)/exp OR (‘depressive’ OR 'depress*' OR 'anxi*' OR 'ptsd' OR 'post-traumatic' OR 'post traumatic' OR 'traumatic stress*' OR 'suicidal behavior' OR ‘suicid*’ OR ‘schizo*’ OR ‘psychotic’ OR ‘psychoses’ OR ‘Self-harm’ OR ‘Self-injur*’ OR ‘deliberate self-harm’ OR ‘parasuicide’ OR ‘self-wounding’ OR ‘self-mutilation’ OR ‘auto-aggression’):ab,ti

#1 and #2

Supplementary Table 2: Classification of included studies according to the pornography-related construct assessed and measurement instrument used

**Author and Year of Publication**	**How was pornography consumption/addiction assessed**	**Construct**
Altin 2024	CYPAT	Pornography addiction / problematic use
Bibi 2024	PCQ and BPS	Problematic pornography use
Borgogna 2018	PPUS	Problematic pornography use
Camilleri 2020	mCIUS	Compulsive pornography use
Doornwaard 2016	Compulsive Internet Use Scale	Compulsive pornography use
Grubbs (MTurk) 2015	Study specific 0-12 scale to quantify consumption, CPUI-9 for pornography addiction	General consumption + self-perceived addiction
Grubbs (Universities) 2015	Study specific 0-12 scale to quantify consumption, CPUI-9 for addiction	General consumption + self-perceived addiction
Guidry 2020	Study specific 5-point Likert scale to quantify consumption	General pornography consumption
Kohut (Zagreb) 2018	Study specific 1-7 scale to quantify consumption	General pornography consumption
Kohut (Rijeka) 2018	Study specific 1-7 scale to quantify consumption	General pornography consumption
Ma 2019	Study specific 5-point Likert scale to quantify consumption	General pornography consumption
Maddock 2019	Excessive Use Subscale of the Problematic Pornography Use Scale and study specific questions	Problematic pornography use
Mattebo 2018	Study specific 0-6 scale to quantify consumption	General pornography consumption

Noel 2023	PPCS-6	Problematic pornography consumption
Sallie 2021	CYPAT	Pornography addiction / problematic use
Varod 2024	PPCS—Short Version	Problematic pornography use
Weaver 2011	SEMB	General pornography consumption
Whitfield 2018	Study specific questionnaire to quantify consumption	General pornography consumption
Willoughby 2019	Study specific questionnaire to quantify consumption	General pornography consumption
Stulhofer 2019	Study specific questionnaire to quantify consumption	General pornography consumption
Nashwa 2025	PAST	Pornography addiction

Abbreviations: CYPAT, Cyber Pornography Addiction Test; PCQ, Pornographic Craving Questionnaire; BPS, Brief Pornography Screening; PPUS, Problematic Pornography Use Scale; mCIUS, Modified Compulsive Internet Use Scale; CPUI-9, Cyber Pornography Use Inventory-9; PPCS-6, Problematic Pornography Consumption Scale-6; PPCS- Short Version, Problematic Pornography Consumption Scale-Short Version; SEMB, Sexually Explicit Media Use Behavior; PAST, Pornography Addiction Screen Tool

Supplementary Table 3: Subgroup meta-analyses of anxiety correlation observed in pornographic content consumers using the random effect model

**Variable**	**Cohorts**	**Number at risk**	**Correlation**	**95%CI**	**I2**	**Test of interaction (p-value)**
Overall	7	4412	0.16	0.08; 0.25	87	NA
WHO Region=AMR	4	2972	0.14	0.08; 0.21	62	0.8647
WHO Region=EMR	2	1108	0.23	-0.08; 0.50	95	
WHO Region=EUR	1	332	0.14	0.03; 0.24	NA	
High income	5	3304	0.14	0.10; 0.19	50	0.6021
Lower-middle income	2	1108	0.23	-0.08; 0.50	95	
Percentage of Males<50%	4	2204	0.19	0.05; 0.33	91	0.4716
Percentage of Males≥50%	3	2208	0.14	0.10; 0.18	6	
Age≤25	4	3132	0.22	0.12; 0.33	89	**0.0321**
Age>25	3	1280	0.09	0.02; 0.15	22	
Scale=DASS	5	2484	0.17	0.04; 0.29	90	0.7503
Scale=GAD-7	2	1928	0.15	0.10; 0.19	0	

Abbreviations: NA, not applicable; CI, Confidence interval; DASS, Depression Anxiety Stress Scale; GAD-7, General Anxiety Disorder-7; WHO, World Health Organisation; AMR, Americas; EMR, Eastern Mediterranean Region; EUR, European Region

Supplementary Table 4: Subgroup meta-analyses of depression correlation among pornographic content consumers using the random effect model

Variable	Cohorts	Number at risk	Correlation	95%CI	I2	Test of interaction (p-value)
Overall	10	6464	0.24	0.15; 0.32	91	NA
Age≤25	6	4864	0.24	0.15; 0.33	90	0.9608
Age>25	4	1600	0.24	0.04; 0.41	93	
Percentage of Males<50%	4	2204	0.23	0.06; 0.39	94	0.9521
Percentage of Males≥50%	6	4260	0.24	0.14; 0.34	87	
High income	7	3955	0.20	0.10; 0.29	84	**0.0060**
Lower-middle income	2	1108	0.40	0.29; 0.50	70	
Upper-middle income	1	1401	0.20	0.15; 0.25	NA	
WHO Region=AMR	5	3292	0.19	0.05; 0.32	89	0.0169
WHO Region=EMR	2	1108	0.40	0.29; 0.50	70	
WHO Region=EUR	2	663	0.22	0.14; 0.29	0	
WHO Region=WPR	1	1401	0.20	0.15; 0.25	NA	

Scale=DASS	5	2484	0.25	0.12; 0.38	92	**<0.0001**
Scale=Depressive Mood Scale	1	331	0.20	0.09; 0.30	NA	
Scale=CES-D-10	2	1928	0.14	0.05; 0.23	73	
Scale=PHQ-9	1	1401	0.20	0.15; 0.25	NA	
Scale=CESD-R-10	1	320	0.44	0.35; 0.52	NA	

Abbreviations: NA, not applicable; CI, Confidence interval; DASS, Depression Anxiety Stress Scale; CES-D-10, Center for Epidemiological Studies Depression Scale-10 item; PHQ-9, Patient Health Questionnaire-9; WHO, World Health Organisation; AMR, Americas; EMR, Eastern Mediterranean Region; EUR, European Region; WPR, Western Pacific Region

Supplementary Table 5: Subgroup meta-analyses of depression scores observed in pornographic content consumers between males and females using the random effect model

Variable	Cohorts	Number of exposed	Number of controls	SMD	95%CI	I2	Test of interaction (p-value)
Overall	5	1824	1811	-0.18	-0.37; 0.02	88	NA
Age≤25	3	1485	1550	-0.19	-0.53; 0.14	94	0.8003
Age>25	2	339	261	-0.15	-0.31; 0.02	0	
Percentage of Males<50%	1	250	507	0.09	-0.06; 0.25	NA	**0.0045**
Percentage of Males≥50%	4	1574	1304	-0.25	-0.43; -0.07	82	
High income	2	727	907	-0.20	-0.78; 0.38	97	0.8234
Lower-middle income	1	164	116	-0.09	-0.33; 0.14	NA	
Upper-middle income	1	758	643	-0.17	-0.28; -0.07	NA	
WHO Region=AMR	1	250	507	0.09	-0.06; 0.25	NA	**<0.0001**
WHO Region=EMR	1	164	116	-0.09	-0.33; 0.14	NA	
WHO Region=EUR	1	477	400	-0.50	-0.63; -0.36	NA	
WHO Region=WPR	1	758	643	-0.17	-0.28; -0.07	NA	

Scale=DASS	2	414	623	0.02	-0.15; 0.20	40	**<0.0001**
Scale=PHQ-9	1	758	643	-0.17	-0.28; -0.07	NA	
Scale=CESD-R-10	1	175	145	-0.19	-0.41; 0.03	NA	
Scale=DSRS	1	477	400	-0.50	-0.63; -0.36	NA	

Abbreviations: NA, not applicable; CI, Confidence interval; SMD; Standardised Mean Difference; DASS, Depression Anxiety Stress Scale; PHQ-9, Patient Health Questionnaire-9; CESD-R-10, Center for Epidemiologic Studies Depression Scale Revised-10 item; DSRS, Depression Self-Rating Scale; WHO, World Health Organisation; AMR, Americas; EMR, Eastern Mediterranean Region; EUR, European Region; WPR, Western Pacific Region

Supplementary Table 6: Evaluation of the mediating or confounding effect of age on pornographic consumption and mental health outcomes

**Author**	**Year**	**Country**	**Study population**	**Key findings†**
Sallie	2021	United Kingdom	1,220 male adolescents, with a mean age of 14.7 years, were recruited from schools across 20 Croatian counties as part of a larger longitudinal study on adolescents' sexual development.	Age was negatively associated with increased amounts of weekly pornographic viewing (rs = -0.29, p = .004) during quarantine.
Varod	2024	Israel	463 participants, with a mean age of 25.41 years (SD = 6.54), were recruited from an anonymous online survey in Israel	The "no/low-frequency non-PPU without sexual problems" group are older (M = 25.41, SD = 6.98) compared to the "high-frequency PPU with sexual problems" group (M = 24.63, SD = 3.77; t = −3.03, SE = 0.03, p = 0.002).
Weaver	2011	USA	559 participants were recruited from a survey of Internet-using adults in Seattle-Tacoma.	Younger respondents are more likely to engage in pornographic consumption compared to older respondents (OR 0.97, 95% CI 0.95–0.99).
Noel	2023	USA	1,022 participants were recruited from the Rhode Island Young Adult Survey in Rhode Island.	Significantly increased risk of pornographic usage with each one year increase in age (OR: 1.14, 95%CI: 1.06-1.22).
Camilleri	2020	USA	1,031 participants were recruited from Franciscan University of Steubenville, Steubenville, Ohio.	Significant association of age, in relation to pornographic use and depression (β=-0.510, p=0.021), but not anxiety (β=-0.355, p=0.071)

Abbreviations: SD, Standard deviation; OR, Odds ratio; CI, Confidence interval; PPU, Problematic pornography use

†Outcomes of interest include logistic or linear regression analysis for any association between age and pornographic consumption

Supplementary Table 7: Evaluation of the mediating or confounding effect of gender on pornographic consumption and mental health outcomes

**Author**	**Year**	**Country**	**Study population**	**Key findings†**
Sallie	2021	United Kingdom	1,220 male adolescents, with a mean age of 14.7 years, were recruited from schools across 20 Croatian counties as part of a larger longitudinal study on adolescents' sexual development.	Males show a greater increase (0.093 ± 5.79 hours, range 0–75) in pornographic viewing compared to females (0.06 ± 1.32 hours, range 0–12.75), p < .0001.
Varod	2024	Israel	463 participants, with a mean age of 25.41 years (SD = 6.54), were recruited from an anonymous online survey in Israel	There are higher proportion of males in the "high-frequency PPU with sexual problems" (47.1%, t = 7.35, p < 0.0001) and "high-frequency non-PPU with low/no sexual problems" (44.9%, t = 6.97, p < 0.0001) groups compared to the "no/low-frequency non-PPU without sexual problems" group (6.5%).
Weaver	2011	USA	559 participants were recruited from a survey of Internet-using adults in Seattle-Tacoma.	Men (78%) are more likely than women (22%) to report pornographic consumption (OR = 7.99, 95% CI: 5.4–11.9).
Štulhofer	2019	Croatia	1,289 Croatian high school students, with a mean age at baseline of 15.9 years (SD = 0.52), were recruited from 14 secondary schools.	Compared to their female peers, male participants reported significantly higher frequency of pornography use (p < 0.001).
Noel	2023	USA	1,022 participants were recruited from the Rhode Island Young Adult Survey in Rhode Island.	Significantly increased risk of pornographic use among males (OR: 4.95, 95%CI: 3.18-7.71), and sexual gender minority (OR: 2.67, 95%CI: 2.02-3.54), compared to females.<br><br>Significantly increased risk of pornographic addiction among males (OR: 13.4, 95%CI: 5.71-31.4) and sexual gender minority (OR: 3.67, 95%CI: 1.64-8.22).
Mattebo	2018	Sweden	1,699 Swedish adolescents, with a mean age of 15.3 years (SD = 0.8), were recruited from 14 schools in two municipalities in a county in Sweden.	Significantly decreased risk of pornographic consumption among females (Mean - 1.72, SD - 1.02) compared to males (Mean - 4.25, SD - 1.31) (p <0.001)
Ma	2019	China	1,401 early Chinese adolescents, with a mean age of 12.43 years (SD = 0.70) ,	Significantly increased risk of pornographic use among males (mean - 0.158, compared to females (0.100) (mean difference, 0.058, t = 2.78, p <0.01).

			were recruited from 13 secondary schools in Hong Kong	
Maddock	2019	Nil (Online)	320 adult individuals, with a mean age of 36.26 years (SD = 10.18), were recruited from MTurk via TurkPrime.com for a 6-month longitudinal study.	Significant association in religiosity and frequency of pornographic use in males (b = 0.27, 95%CI: 0.06-0.49) but not females (b = 0.06, 95%CI: -0.29-0.17)

Abbreviations: SD, Standard deviation; OR, Odds ratio; CI, Confidence interval; PPU, Problematic pornography use

†Outcomes of interest include logistic or linear regression analysis for any association between gender and pornographic consumption

Supplementary Table 8: Evaluation of the mediating or confounding effect of religiosity on pornographic consumption and mental health outcomes

**Author**	**Year**	**Country**	**Study population**	**Key findings†**
Ma	2018	China	1,401 early Chinese adolescents, with a mean age of 12.43 years (SD = 0.70), were recruited from 13 secondary schools in Hong Kong	No significant difference in pornographic consumption between those who are religious (mean = 0.09) and those who are not religious (mean = 0.13) (mean difference 0.04, t = -1.66, p >0.05).
Maddock	2019	Nil	320 adult individuals, with a mean age of 36.26 years (SD = 10.18), were recruited from MTurk via TurkPrime.com for a 6-month longitudinal study.	No significant association in self-perceived pornographic use and religiosity (r = -0.34, p > 0.05) in both males (b = 0.34, 95%CI: -1.04-0.36) and females (b = 1.06, 95% CI: -1.76-3.87).<br><br>Significant association in religiosity and frequency of pornographic use in males (b = 0.27, 95%CI: 0.06-0.49) but not females (b = 0.06, 95%CI: -0.29-0.17)

Abbreviations: SD, Standard deviation; CI, Confidence interval

†Outcomes of interest include logistic or linear regression analysis for any association between religiosity and pornographic consumption

Supplementary Table 9: Evaluation of the mediating or confounding effect of self-esteem on pornographic consumption and mental health outcomes

**Author**	**Year**	**Country**	**Study population**	**Key findings†**
Kohut	2018	Croatia	2 samples were collected.<br><br>Sample 1 (PROBIOPS Study): 1,011 adolescents, with a mean age of 14.7 years (SD = 0.7), were recruited from Croatian secondary schools.<br><br>Sample 2 (German Sample): 654 adolescents, with a mean age of 14.8 years (SD = 0.5), were recruited from a German longitudinal study.	No significant association was found between pornographic consumption and self-esteem in both Zagreb (b = 0.01, p = 0.924) and Rijeka (b = 0.05, p = 0.153)
Doornward	2016	Netherlands	331 Dutch boys, with a mean age of 15.16 years, were included in the study.	Significantly increased risk of compulsive pornographic use in those with low global self-esteem (B = -0.44, p <0.01, RR = 0.65, 95%CI: 0.43-0.86)

Abbreviations: SD, Standard deviation; RR, Risk ratio; CI, Confidence interval

†Outcomes of interest include logistic or linear regression analysis for any association between self-esteem and pornographic consumption

Supplementary Table 10: Quality assessment of included cohort studies using the Joanna Brigg's Institute Critical Appraisal tool

**Study**	**1**	**2**	**3**	**4**	**5**	**6**	**7**	**8**	**9**	**10**	**11**
Doornwaard	**Y**	**Y**	**Y**	**Y**	**Y**	**N**	**Y**	**Y**	**Y**	**N**	**Y**
Grubbs	**Y**	**Y**	**Y**	**Y**	**Y**	**N**	**Y**	**Y**	**U**	**N**	**Y**
Kohut	**Y**	**Y**	**Y**	**Y**	**Y**	**N**	**Y**	**Y**	**Y**	**Y**	**Y**
Ma	**Y**	**Y**	**Y**	**Y**	**Y**	**N**	**Y**	**Y**	**Y**	**Y**	**Y**
Maddock	**NA**	**NA**	**Y**	**Y**	**Y**	**N**	**Y**	**Y**	**N**	**N**	**Y**
Mattebo	**Y**	**Y**	**Y**	**Y**	**Y**	**N**	**Y**	**Y**	**N**	**N**	**Y**
Whitfield	**N**	**N**	**Y**	**Y**	**Y**	**N**	**Y**	**Y**	**N**	**N**	**Y**
Stulhofer	**Y**	**Y**	**Y**	**Y**	**Y**	**N**	**Y**	**Y**	**Y**	**Y**	**Y**

Checklist
1. Were the two groups similar and recruited from the same population?
2. Were the exposures measured similarly to assign people to both exposed and unexposed groups?
3. Was the exposure measured in a valid and reliable way?
4. Were confounding factors identified?
5. Were strategies to deal with confounding factors stated?
6. Were the groups/participants free of the outcome at the start of the study (or at the moment of exposure)?
7. Were the outcomes measured in a valid and reliable way?
8. Was the follow up time reported and sufficient to be long enough for outcomes to occur?

9. Was follow up complete, and if not, were the reasons to loss to follow up described and explored?
10. Were strategies to address incomplete follow up utilized?
11. Was appropriate statistical analysis used?

Legend:
Y – Yes
N – No
U – Unclear
NA – Not applicable

Supplementary Table 11: Quality assessment of included cross-sectional studies using the Joanna Briggs Institute Critical Appraisal tool

**Study**	**1**	**2**	**3**	**4**	**5**	**6**	**7**	**8**
Altin	**Y**	**Y**	**Y**	**Y**	**Y**	**Y**	**Y**	**Y**
Bibi	**Y**	**Y**	**Y**	**Y**	**Y**	**Y**	**Y**	**Y**
Borgogna	**Y**	**Y**	**Y**	**Y**	**Y**	**Y**	**Y**	**Y**
Camilleri	**Y**	**Y**	**Y**	**Y**	**Y**	**Y**	**Y**	**Y**
Guidry	**Y**	**Y**	**Y**	**Y**	**Y**	**Y**	**Y**	**Y**
Noel	**Y**	**Y**	**Y**	**Y**	**Y**	**Y**	**Y**	**Y**
Sallie	**Y**	**Y**	**Y**	**Y**	**Y**	**Y**	**Y**	**Y**
Varod	**Y**	**Y**	**Y**	**Y**	**Y**	**Y**	**Y**	**Y**
Weaver	**Y**	**Y**	**Y**	**Y**	**Y**	**Y**	**Y**	**Y**
Willoughby	**Y**	**Y**	**Y**	**Y**	**Y**	**Y**	**Y**	**Y**
Nashwa	**Y**	**Y**	**Y**	**Y**	**Y**	**Y**	**Y**	**Y**

Checklist
1. Were the criteria for inclusion in the sample clearly defined?
2. Were objective, standard criteria used for measurement of the condition?
3. Was the exposure measured in a valid and reliable way?
4. Were the outcomes measured in a valid and reliable way?
5. Were confounding factors identified?

6. Were strategies to deal with confounding factors stated?
7. Was appropriate statistical analysis used?
8. Were the study subjects and the setting described in detail?

Legend:
Y – Yes
N – No
U – Unclear
NA – Not applicable

Supplementary Figure 1: Pooled correlation scores between pornography consumption and anxiety, subgrouped by consumption habits.

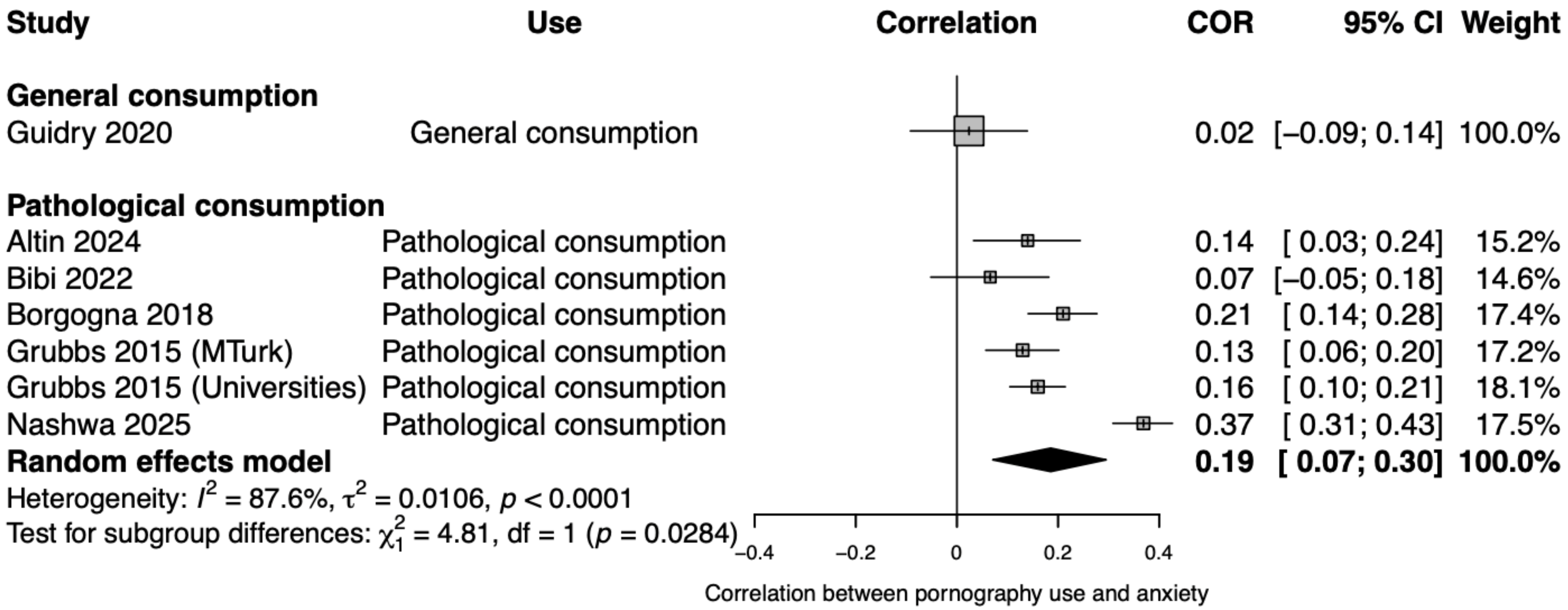

Supplementary Figure 2: Pooled correlation scores between pornography consumption and depression, subgrouped by consumption habits.

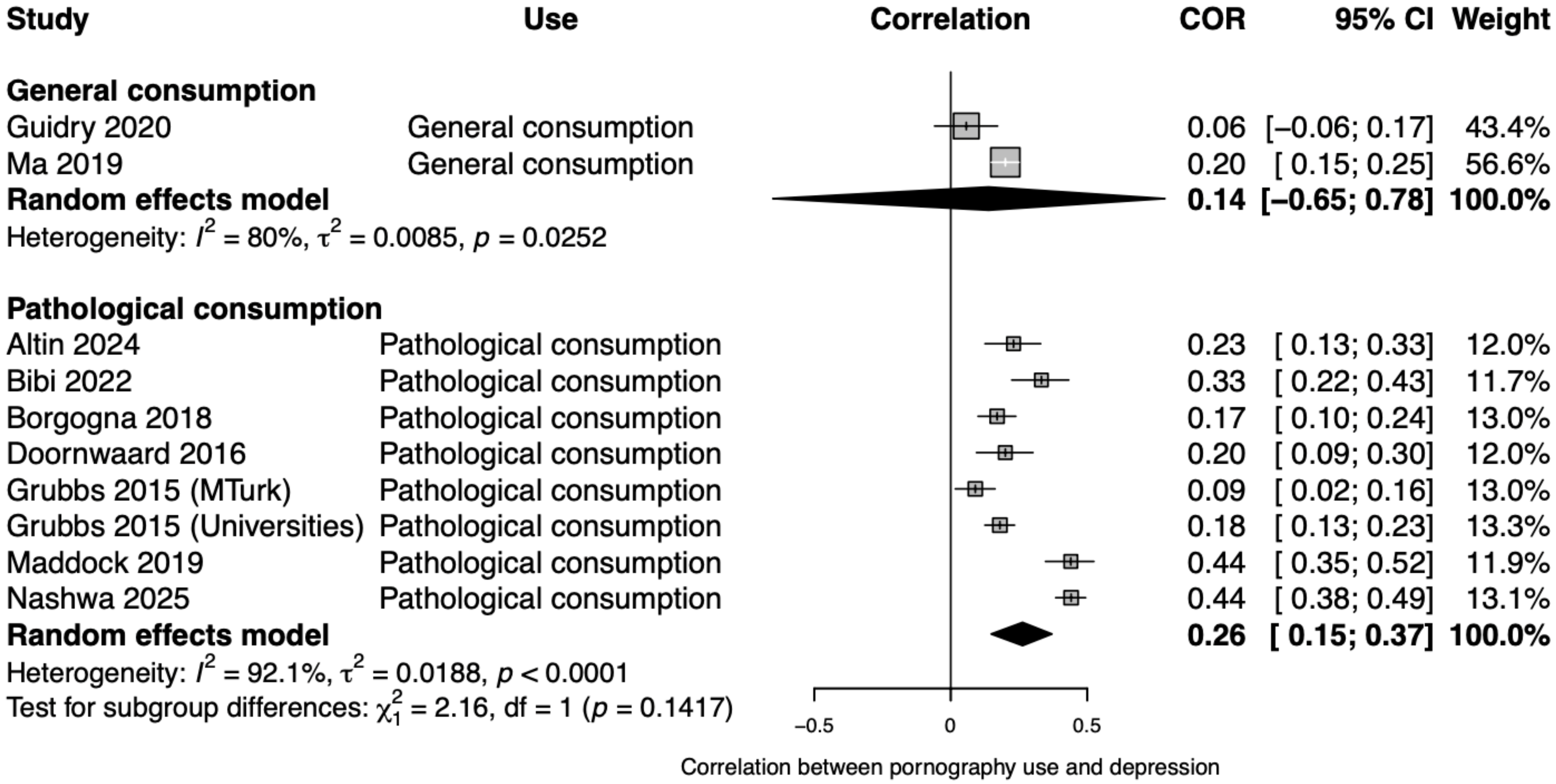

Supplementary Figure 3: Outlier assessment of studies assessing depression correlation among pornographic content consumers using the random effects model

```
Identified outliers (random-effects model)
------------------------------------------
"Maddock 2019"

Results with outliers removed
-----------------------------
Number of studies: k = 8
Number of observations: o = 5316

                        COR           95%-CI    z  p-value
Random effects model 0.1803 [0.1299; 0.2298] 6.91 < 0.0001

Quantifying heterogeneity (with 95%-CIs):
 tau^2 = 0.0036 [0.0005; 0.0289]; tau = 0.0601 [0.0224; 0.1701]
 I^2 = 64.1% [23.1%; 83.2%]; H = 1.67 [1.14; 2.44]

Test of heterogeneity:
     Q d.f. p-value
 19.48    7  0.0068

Details of meta-analysis methods:
- Inverse variance method
- Restricted maximum-likelihood estimator for tau^2
- Q-Profile method for confidence interval of tau^2 and tau
- Calculation of I^2 based on Q
- Fisher's z transformation of correlations
```

Supplementary Figure 4: Leave-one-out assessment of studies assessing depression correlation among pornographic content consumers using the random effects model

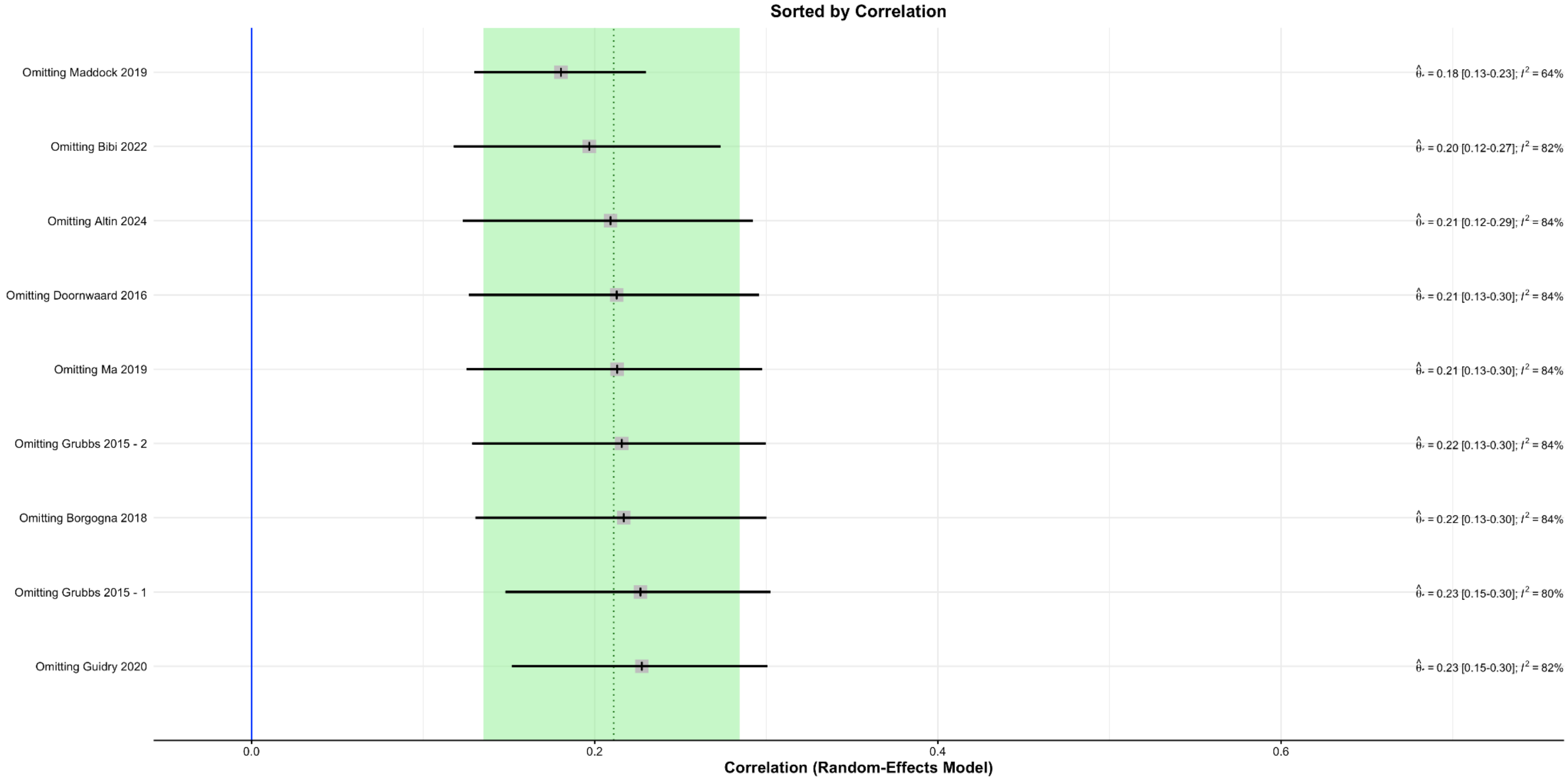

Supplementary Figure 5: Outlier assessment of studies assessing anxiety correlation among pornographic content consumers using the random effects model

```
Identified outliers (random-effects model)
------------------------------------------
"Nashwa 2025"

Results with outliers removed
-----------------------------
Number of studies: k = 6
Number of observations: o = 3584

                        COR           95%-CI    z  p-value
Random effects model 0.1349 [0.0861; 0.1831] 5.38 < 0.0001

Quantifying heterogeneity (with 95%-CIs):
 tau^2 = 0.0018 [0.0000; 0.0251]; tau = 0.0430 [0.0000; 0.1586]
 I^2 = 49.3% [0.0%; 79.9%]; H = 1.40 [1.00; 2.23]

Test of heterogeneity:
    Q d.f. p-value
 9.86    5  0.0794

Details of meta-analysis methods:
- Inverse variance method
- Restricted maximum-likelihood estimator for tau^2
- Q-Profile method for confidence interval of tau^2 and tau
- Calculation of I^2 based on Q
- Fisher's z transformation of correlations
```

Supplementary Figure 6: Leave-one-out assessment of studies assessing anxiety correlation among pornographic content consumers using the random effects model

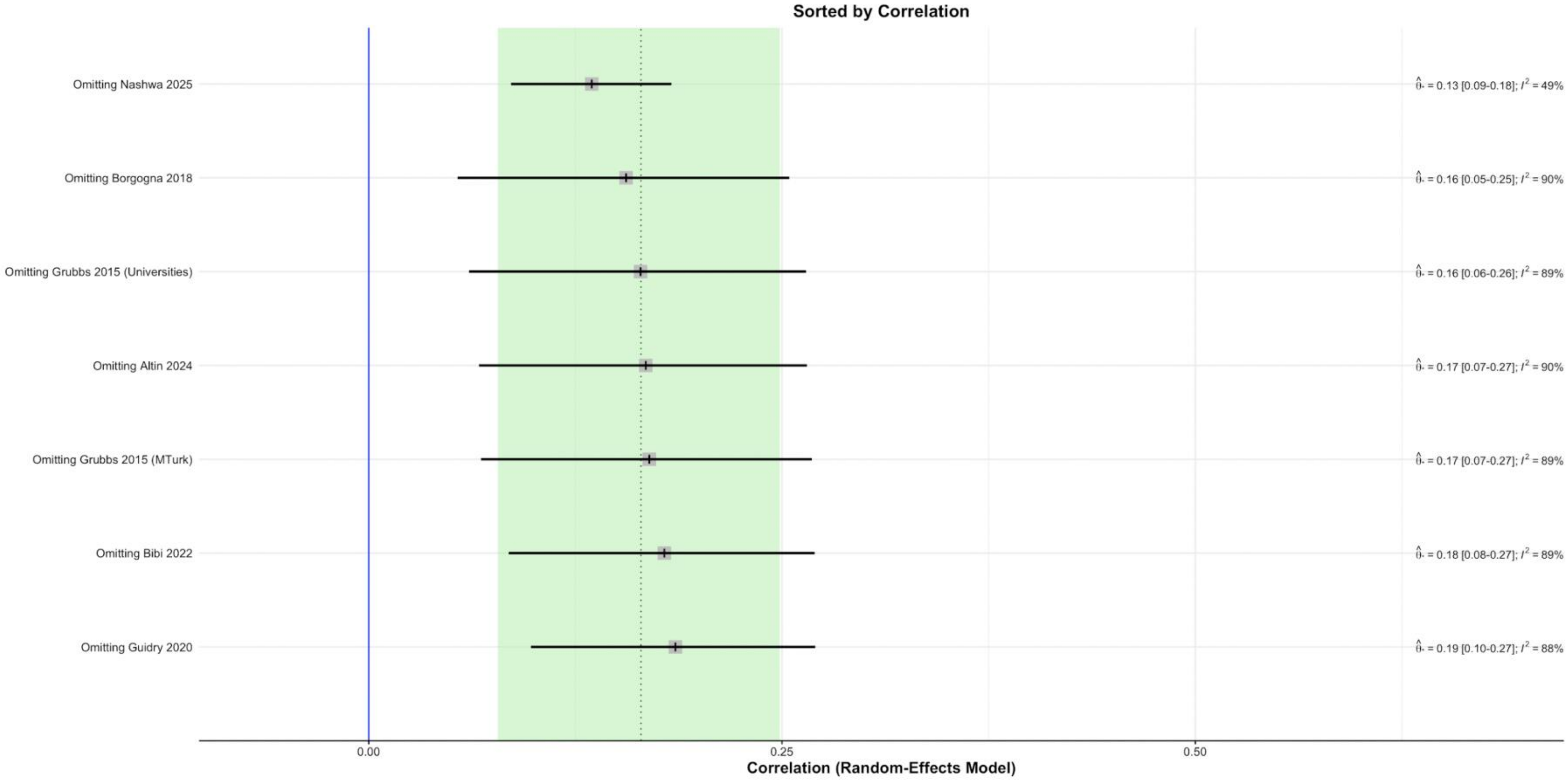

Supplementary Figure 7: Outlier assessment of studies assessing depression scores among pornographic content consumers between males and females using the random effects model

No outliers detected (random-effects model).

Supplementary Figure 8: Leave-one-out assessment of studies assessing depression scores among pornographic content consumers between males and females using the random effects model

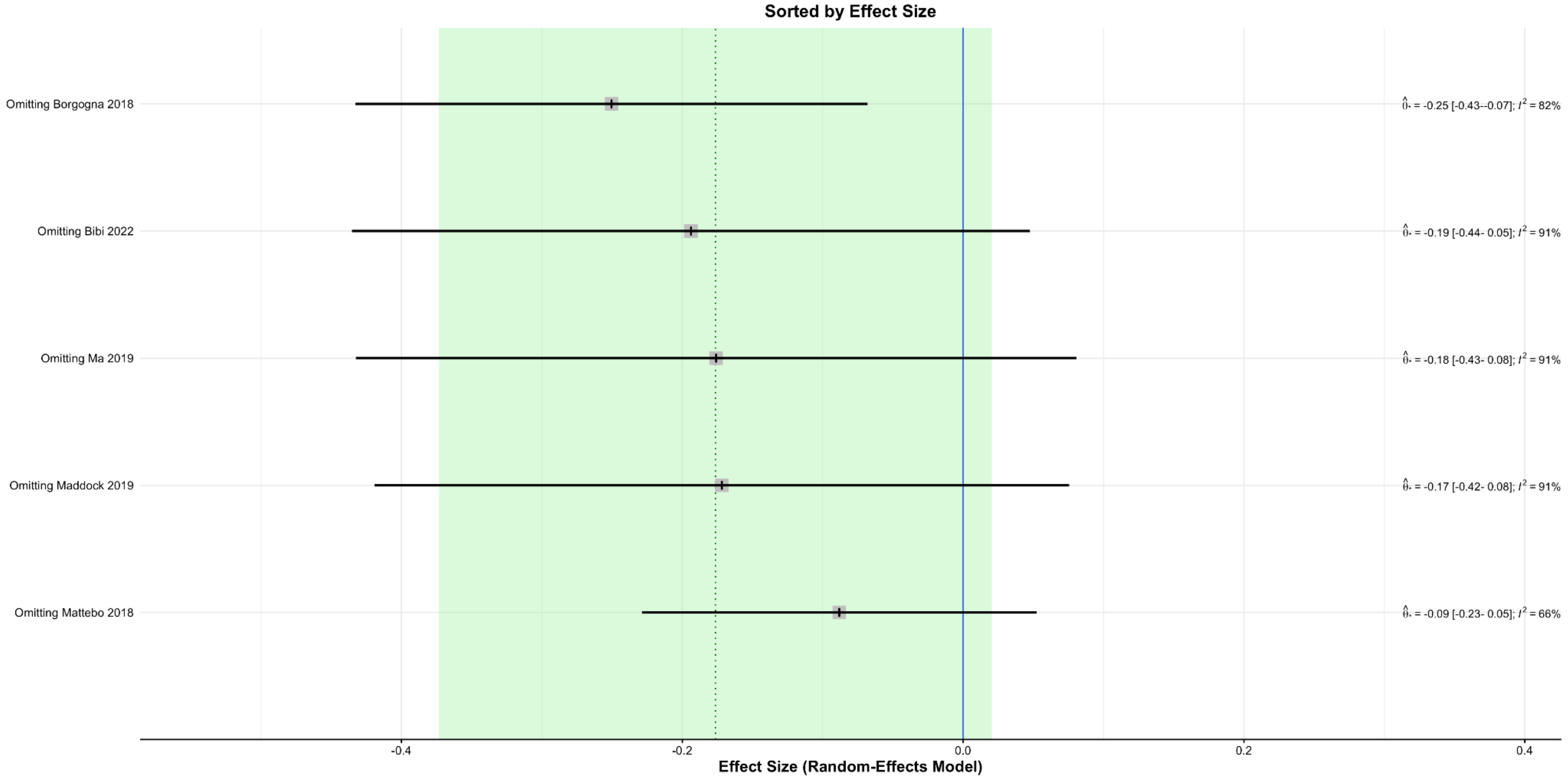

Supplement: Supplementary file 1 [file jcm-15-05030-s001.zip › jcm-4325148-supplementary.pdf]
